# Clinical Factors Associated with 90-Day Mortality in Patients Receiving Colistin Therapy for Multidrug-Resistant Gram-Negative Periprosthetic Joint Infection

**DOI:** 10.3390/jcm15072759

**Published:** 2026-04-06

**Authors:** Bahattin Kemah, Alper Dünki, Ömer Polat, Özkan Öztürk, Furkan Uçar, Savaş Çamur, Çağrı Özcan

**Affiliations:** Department of Orthopaedics and Traumatology, Umraniye Training and Research Hospital, Health Sciences University, 34764 Istanbul, Türkiye; bahattinkemah.md@gmail.com (B.K.); alperdunki93@yahoo.com (A.D.); dromerpolat@gmail.com (Ö.P.); ozkanozturkmd@gmail.com (Ö.Ö.); furkanuar29@gmail.com (F.U.); savascamur@gmail.com (S.Ç.)

**Keywords:** periprosthetic joint infection, Gram-negative, colistin, ASA, mortality, MDR

## Abstract

**Objectives:** Colistin is frequently used as a last-line treatment option for periprosthetic joint infections (PJIs) caused by multidrug-resistant (MDR) Gram-negative pathogens, and mortality rates are high in this patient group. This study aimed to evaluate 90-day mortality and its associated clinical factors in MDR Gram-negative PJI cases treated with colistin, with particular attention to the American Society of Anesthesiologists (ASA) score and changes in renal function during therapy. **Methods:** Patients diagnosed with MDR Gram-negative PJI and treated with intravenous colistin at a single center between 2010 and 2024 were retrospectively reviewed. Demographic data, infection localization, comorbidities, American Society of Anesthesiologists (ASA) score, causative pathogens, presence of sepsis, intensive care unit (ICU) admission, duration of colistin therapy, pre- and post-treatment renal function parameters, and mortality were all recorded. Survivors and non-survivors were compared using univariate analysis. **Results:** The cohort included 44 patients with a mean age of 72 years (23 women and 21 men). Infections involved the hip in 33 patients (75.0%) and the knee in 11 (25.0%). Twenty-two patients (50.0%) were in the high-ASA group (ASA ≥ III group). Within 90 days of initiating colistin therapy, 25 patients died and 19 survived. A high ASA score was significantly more common among non-survivors than among survivors (18/25 (72.0%) vs. 4/19 (21.1%); *p* < 0.001), and dialysis requirement was also more common among non-survivors [20/25 (80.0%) vs. 8/19 (42.1%); *p* = 0.013]. End-of-treatment renal parameters were significantly worse among non-survivors, including urea [71.2 (50.9–78.8) vs. 38.5 (34.8–42.5) mg/dL; *p* = 0.003], creatinine [2.29 (1.75–2.64) vs. 0.93 (0.72–1.60) mg/dL; *p* = 0.003], urea delta [42.8 (38.0–48.6) vs. −5.4 (−7.9 to −2.0) mg/dL; *p* = 0.006], and creatinine delta [0.78 (0.33–1.57) vs. 0.16 (0.10–0.57) mg/dL; *p* = 0.008] levels. In contrast, age, sepsis, ICU admission, and colistin treatment duration were not significantly associated with 90-day survival. **Conclusions:** In this high-risk cohort of patients with MDR Gram-negative PJI treated with colistin, the 90-day mortality rate was high and was associated with higher American Society of Anesthesiologists scores, dialysis requirements, and worsening renal function during treatment. No significant association was observed between treatment duration and response in this cohort. These findings should be interpreted cautiously, given the retrospective design and the limited sample size.

## 1. Introduction

Periprosthetic joint infection (PJI) is one of the most serious complications of total hip and knee arthroplasty and represents a substantial burden in terms of morbidity and mortality. The reported incidence is 1–2% after primary arthroplasty and may reach 4% after revision procedures [1,2]. Successful PJI management depends on multiple clinical variables, including the patient’s overall condition, timing of infection onset, surgical approach, and antimicrobial susceptibility of the causative microorganism [3,4].

In recent years, an increase in Gram-negative bacteria, particularly multidrug-resistant (MDR) and extensively drug-resistant (XDR) strains, has been reported as a cause of PJI [5,6]. Infections caused by pathogens such as *Acinetobacter baumannii*, *Klebsiella pneumoniae*, and *Pseudomonas aeruginosa* have become increasingly important clinical problems because of limited treatment options, high recurrence rates, and increased mortality risks [6,7,8]. Contemporary series have shown substantially higher rates of treatment failure and mortality in MDR/XDR Gram-negative PJI [6,8,9].

In MDR/XDR Gram-negative PJI, colistin (polymyxin E) is often one of the last options for intravenous therapy [6,9,10]. However, its considerable nephrotoxicity poses additional risks, particularly in elderly patients, those with a high comorbidity burden, and those with hemodynamic instability [10,11,12]. Although clinical observations and some cohort studies report high mortality among patients receiving colistin, the extent to which mortality is attributable to drug toxicity versus accompanying clinical factors (high ASA score, sepsis and renal impairment) remains unclear [6,11].

The American Society of Anesthesiologists (ASA) classification is widely used in the surgical literature to summarize the comorbidity burden and physiological reserve and to predict perioperative morbidity and mortality. Increasing ASA scores have been associated with long-term mortality in both large national registry studies and PJI-focused series [1,2,13]. However, studies that specifically evaluate the independent effect of ASA score on mortality among patients receiving colistin for MDR PJI are limited [6,9].

Therefore, a clinically critical question is whether the primary driver of mortality in resistant Gram-negative PJI treated with colistin is the antibiotic choice or the patient’s systemic condition. We hypothesized that in patients with MDR Gram-negative PJI treated with colistin, 90-day mortality is primarily associated with high ASA scores, advanced age, and renal dysfunction. Accordingly, this study aimed to evaluate the association between ASA scores and 90-day mortality and to identify independent clinical determinants of mortality in cases of MDR Gram-negative PJI treated with colistin.

Patients with MDR Gram-negative PJI who require colistin represent a particularly vulnerable subgroup, in whom mortality may be influenced not only by infection severity but also by baseline physiological reserve and treatment-related toxicity. In this setting, age may reflect reduced systemic resilience, the ASA score may capture the overall comorbidity burden and frailty, and renal dysfunction may represent both baseline vulnerability and a clinically important complication. However, data specifically addressing clinical factors associated with mortality in colistin-treated MDR Gram-negative PJI remain limited. Therefore, this study aimed to evaluate 90-day mortality and its associated clinical factors in this high-risk cohort.

We hypothesized that a higher ASA score, advanced age, and worsening renal function during treatment would be associated with increased 90-day mortality in patients receiving colistin for MDR Gram-negative PJI.

## 2. Materials and Methods

### 2.1. Study Design and Patient Selection

This retrospective cohort analysis included PJI cases with MDR Gram-negative microorganisms identified in cultures, followed up at the Orthopedics and Traumatology Department of Umraniye Training and Research Hospital between 2010 and 2024. Electronic health records, operative notes, and microbiology reports were reviewed to identify patients diagnosed with hip or knee PJI who received intravenous colistin therapy. Written informed consent for participation and the use of clinical data was obtained from all patients and/or their legal guardians at the time of treatment.

The inclusion criteria were age ≥18 years; diagnosis of hip or knee PJI following arthroplasty; isolation of an MDR Gram-negative organism from synovial fluid, tissue culture, or periprosthetic specimens; receipt of intravenous colistin; and availability of at least 90 days of mortality data. Patients who did not meet the PJI diagnostic criteria, had Gram-positive or fungal PJI, had culture-negative PJI, had Gram-negative infections that did not meet the MDR criteria, received colistin for <48 h, or had <90 days of follow-up or missing mortality data were excluded.

### 2.2. Definitions

PJI diagnosis was established jointly by the orthopedic surgery and infectious disease teams based on clinical findings, laboratory parameters, imaging findings when applicable, and microbiological confirmation from intraoperative samples, including the presence of at least two positive cultures in accordance with contemporary diagnostic frameworks. MDR Gram-negative infection was defined as resistance to at least three different antimicrobial classes.

The ASA physical status classification was used as a global measure of the preoperative systemic disease burden. ASA was analyzed both as a continuous variable and, for descriptive and comparative purposes, as a dichotomous variable (ASA < III vs. ASA ≥ III), because ASA III and above generally indicates severe systemic disease and represents a clinically meaningful threshold for increased clinical risk. Further subgrouping was not considered appropriate because of the limited sample size of this study. Sepsis was recorded based on systemic findings consistent with infection at the initiation of colistin therapy. ICU admission was defined as requiring ICU monitoring at any time before or during the colistin therapy.

### 2.3. Colistin Therapy

Colistin was administered intravenously to all patients according to the institutional protocol based on a joint decision by the infectious disease specialists and the orthopedic team. The initial dosing, use of loading dose, maintenance doses, and treatment duration were individualized according to renal function, body weight, and infection severity. The colistin treatment duration (days) was calculated from the date of the first dose to the date of the last dose. The surgical approach (e.g., debridement and implant retention, one-/two-stage revision) was determined by clinical indications. As this study primarily focused on medical therapy and mortality, surgical details were recorded descriptively only.

### 2.4. Data Collection

Hospital records were retrospectively reviewed. The collected data included demographics (age and sex), infection characteristics (affected joint [hip/knee], culture positivity and causative microorganism), comorbidities (diabetes mellitus, hypertension, chronic obstructive pulmonary disease, and heart failure), ASA score, sepsis at colistin initiation, ICU admission, total hospital length of stay (days), duration of colistin therapy (days), serum urea and creatinine levels (baseline and end of treatment), post-treatment urea and creatinine values, urea delta, creatinine delta, dialysis requirement, and all-cause mortality within 90 days after initiation of colistin therapy.

### 2.5. Statistical Analysis

Statistical analyses were performed using SPSS software (version 26.0; IBM Corporation, Armonk, NY, USA). Continuous variable distributions were assessed using visual methods (histograms and probability plots) and normality tests. Normally distributed continuous variables are presented as mean ± standard deviation, and non-normally distributed variables as the median and interquartile range (IQR). Categorical variables are presented as frequencies and percentages (%). Comparisons between survivors and non-survivors were performed using the independent-samples *t*-test or the Mann–Whitney U test, as appropriate. Categorical variables were compared using the chi-square or Fisher’s exact tests, as appropriate. A two-sided *p* < 0.05 was considered to be statistically significant.

Univariate analyses were initially conducted to identify the clinical and laboratory variables associated with 90-day mortality. Subsequently, variables that were statistically significant and clinically relevant were entered into a multivariate logistic regression model to explore their independent associations with mortality. However, given the limited sample size, relatively small number of events, and potential confounding effects of renal function parameters and dialysis requirements, multivariate analysis was considered, and its results were interpreted.

## 3. Results

A total of 44 patients were enrolled in the study. The mean age was 72 years, and 23 patients (52.3%) were women and 21 (47.7%) were men. Infections were associated with hip arthroplasty in 33 patients (75.0%) and knee arthroplasty in 11 (25.0%) patients. Twenty-two patients (50.0%) were classified as having a high ASA (ASA ≥ III).

Ninety days after colistin initiation, 25 patients (56.8%) died and 19 (43.2%) survived. Non-survivors tended to be older than survivors; however, this difference was not statistically significant (*p* = 0.41). There were no significant differences in sex distribution or whether the infection involved the hip or knee prosthesis (all *p* > 0.10).

In the univariable analyses, a higher ASA score, dialysis requirement, and changes in renal function parameters were significantly associated with 90-day mortality. A high ASA score (ASA ≥ III) was significantly more common among non-survivors than among survivors [18/25 (72.0%) vs. 4/19 (21.1%); *p* < 0.001]. Dialysis was required by 20/25 (80.0%) non-survivors and 8/19 (42.1%) survivors (*p* = 0.013).

Regarding renal parameters, both end-of-treatment urea and creatinine levels and delta values were significantly higher in non-survivors. The median end-of-treatment urea level was 38.5 mg/dL (IQR 34.8–42.5) in survivors and 71.2 mg/dL (IQR 50.9–78.8) in non-survivors (*p* = 0.003). The delta urea value was −5.4 mg/dL (IQR, −7.9 to −2.0) in survivors and 42.8 mg/dL (IQR, 38.0–48.6) in non-survivors (*p* = 0.006). Similarly, the median end-of-treatment creatinine level was 0.93 mg/dL (IQR, 0.72–1.60) in survivors and 2.29 mg/dL (IQR, 1.75–2.64) in non-survivors (*p* = 0.003), and the creatinine delta value was 0.16 mg/dL (IQR, 0.10–0.57) and 0.78 mg/dL (IQR, 0.33–1.57), respectively (*p* = 0.008) (Table 1).

In contrast, baseline sepsis [8/19 (42.1%) vs. 12/25 (48.0%); *p* = 0.77], ICU admission [10/19 (52.6%) vs. 16/25 (64.0%); *p* = 0.54], colistin treatment duration [median 18 days (IQR 8–20) vs. 16 days (IQR 8–25); *p* = 0.79], and evaluated comorbidities (diabetes, hypertension, COPD, heart failure) were not significantly associated with 90-day mortality (all *p* > 0.10). No significant association was observed between 90-day mortality and colistin treatment duration in this cohort study.

In multivariate logistic regression analysis, higher ASA (ASA ≥ III) score (OR: 5.30, 95% CI: 1.10–25.61, *p* = 0.038), delta urea (OR: 1.15, 95% CI: 1.02–1.31, *p* = 0.022) were identified as independent risk factors for 90-day mortality. Delta creatinine was not significant in the multivariate analysis.

## 4. Discussion

In this study, clinical determinants associated with 90-day mortality were evaluated in patients who received colistin for PJI caused by MDR Gram-negative pathogens. Our findings demonstrated a high 90-day mortality rate (56.8%) in this population, with mortality being significantly associated with high ASA scores (ASA ≥ III), marked renal dysfunction during treatment (increases in urea/creatinine), and dialysis requirement. In contrast, age, baseline comorbidities, presence of sepsis, ICU admission, and colistin treatment duration were not significantly associated with 90-day survival. These results suggest that in MDR Gram-negative PJI, outcomes may be driven more by physiological reserve and organ function than by the antibiotic regimen itself, consistent with contemporary literature emphasizing the surgical and microbiological challenges in this setting [9].

High mortality rates of MDR Gram-negative PJI have also been reported previously. Limited treatment options, the complexity of surgical revisions, and severe underlying comorbidity profiles hinder infection control and contribute to increased mortality [6,8,14]. The 56.8% 90-day mortality observed in our study is notably higher than the mortality rates reported in classic PJI series and aligns with the elevated mortality trend reported in series focusing on MDR Gram-negative PJI [6,8,9,13]. The tertiary referral nature of our center and the advanced age, high comorbidity burden, and frequent ICU needs of the included patients may partially explain the relatively high mortality rate.

These findings indicate that categorically labelling colistin as an agent that “causes high mortality” may be clinically misleading and should be reconsidered. Colistin use likely represents a surrogate marker of severe infection rather than an isolated pharmacological risk factor. As highlighted in the literature, the high mortality observed among colistin-treated patients is often less related to direct drug toxicity and more to the selected, severely ill risk profile of the population in whom colistin is used [11,14]. In our cohort, the key factors associated with mortality—high ASA score, substantial renal dysfunction, and dialysis requirement—suggest that colistin may serve as a marker of high-risk clinical scenarios. This perspective supports individualized risk–benefit assessment rather than categorical avoidance of colistin, focusing on identifying patients in whom colistin use may still carry an acceptable nephrotoxicity and mortality risk [6,9,10].

The significantly higher frequency of dialysis requirement in the mortality group indicates severe renal deterioration both biochemically and clinically. This underscores the need for careful baseline renal evaluation, early nephrology consultation when feasible, and minimization of nephrotoxic burden (e.g., concomitant nephrotoxic antibiotics, contrast agents) in patients with MDR Gram-negative PJI for whom colistin is planned [10,11,12,15,16]. Close monitoring of urea and creatinine trends during treatment and prompt dose adjustments, consideration of alternative regimens, or reassessment of colistin therapy in cases with marked increases may represent actionable strategies to reduce mortality in this population.

The association between higher ASA scores and mortality may reflect more than just perioperative anesthetic risk. In this cohort, ASA likely functioned as a broader surrogate of frailty, cumulative comorbidity burden, and limited physiologic reserve in the setting of severe infection, repeated surgical intervention, prolonged hospitalization, and exposure to potentially nephrotoxic therapy. Patients with reduced systemic reserves may be less tolerant to the combined burden of infection-related inflammation, metabolic stress, and organ dysfunction.

The observed relationship between renal dysfunction, dialysis requirements, and mortality should be interpreted in a multifactorial context. In patients with MDR Gram-negative PJI, worsening renal function may arise from several overlapping mechanisms, including baseline vulnerability, sepsis-related hypoperfusion, critical illness, and potential nephrotoxic effects of colistin or concomitant treatments. Accordingly, the need for dialysis may represent both a marker of disease severity and an indicator of severe end-organ dysfunction during treatment, rather than a single, isolated causal factor.

In this study, creatinine delta was significantly associated with mortality in univariable analyses but did not retain statistical significance in the multivariable analysis. This difference likely reflects the fact that univariate analyses evaluate each parameter independently, whereas multivariate models account for the combined effects of related variables. In our study, urea and creatinine both represent renal dysfunction and are therefore closely related to each other. When included in the same model, urea better captured the clinical impact of renal deterioration, whereas the additional contribution of creatinine was reduced. Furthermore, the relatively small sample size may have limited the ability of the model to detect independent effects among variables that are closely correlated. Taken together, these findings suggest that urea-related changes may be more informative than creatinine levels in reflecting mortality risk in this patient population.

Notably, some variables anticipated in our hypothesis, such as advanced age and sepsis, were not significantly associated with 90-day mortality. With respect to age, the clustering of our cohort in an overall older age spectrum may have reduced the discriminatory power of age; compared with larger series showing an effect of age on mortality in PJI, this may reflect the sample structure [1,2,13]. Regarding sepsis and ICU admission, the fact that most patients referred to our center already presented with severe clinical pictures may have limited the ability of these variables to discriminate between mortality. Contemporary reviews emphasize that MDR Gram-negative PJI often follows a severe clinical course with pronounced surgical and microbiological difficulties, with mortality shaped by the combined effects of multiple factors [9]. In our series, the lack of a significant association between sepsis/ICU admission and mortality suggests that while these parameters remain part of a high-risk environment, more specific markers, such as ASA score and renal function, may better reflect mortality risk.

This study has several clinically relevant implications. First, a systematic assessment of ASA score and baseline renal function in patients with MDR Gram-negative PJI for whom colistin is planned may facilitate early identification of high-risk patients [1,2,13]. Second, close monitoring of urea/creatinine trends and implementation of aggressive nephroprotective strategies in cases with significant increases may help prevent progression to renal failure and thereby reduce mortality [10,11,12,15,16]. Third, when non-colistin options are appropriate, more selective planning of colistin use may be rational in patients with limited renal reserve and high ASA scores [10]. Similar mortality patterns may be expected in other severely ill patients receiving colistin for MDR infections beyond orthopedic settings.

### Limitations

This study had several limitations. First, its retrospective and single-center design limits causal inference and increases the possibility of selection bias in the study. Second, the relatively small sample size limited the statistical power and restricted the ability to adequately control for confounding variables; therefore, the observed findings should be interpreted as exploratory associations rather than definitive independent predictors. Third, the study period extended from 2010 to 2024, during which diagnostic approaches to PJI, ICU supportive care, renal monitoring strategies, antimicrobial stewardship, and colistin use threshold may have evolved. This temporal variability may have introduced treatment heterogeneity and unmeasured confounding factors. Fourth, colistin dosing strategies, concomitant antibiotic regimens, and surgical approaches were individualized, and this heterogeneity could not be fully controlled in mortality analyses. Finally, renal dysfunction was evaluated using urea/creatinine levels and dialysis requirements, although detailed acute kidney injury staging was not available.

Overall, in patients with MDR Gram-negative PJI treated with colistin, 90-day mortality appears to be largely determined by baseline systemic physiological reserve (ASA score) and renal dysfunction that develops during therapy. These results indicate that selecting an appropriate antibiotic regimen alone is insufficient, and an individualized, nephrotoxicity-focused, multidisciplinary approach tailored to physiological capacity is essential. Larger prospective multicenter studies are needed to validate these findings and develop algorithmic treatment strategies based on risk stratification in MDR Gram-negative PJI.

## 5. Conclusions

In this high-risk cohort of patients treated with colistin for MDR Gram-negative PJI, 90-day mortality was high and was associated with higher ASA scores, worsening renal function during treatment, and the need for renal replacement therapy. No significant association was observed between treatment duration and treatment response in this cohort. Given the retrospective design and limited sample size, these findings should be interpreted cautiously and confirmed in larger, multicenter studies.

## Figures and Tables

**Table 1:** Comparison of patient characteristics according to 90-day mortality status.

Variable	Survived (*n* = 19)	Died (*n* = 25)	*p*-Value
Age, years	71.3 ± 6.6	72.5 ± 8.6	0.41 ^a^
Female sex, *n* (%)	8 (42.1)	15 (60.0)	0.36 ^b^
Hip PJI, *n* (%)	12 (63.2)	21 (84.0)	0.16 ^b^
ASA score	2.05 ± 0.62	2.72 ± 0.46	<0.001 ^c^
ASA ≥ 3, *n* (%)	4 (21.1)	18 (72.0)	<0.001 ^b^
Dialysis, *n* (%)	8 (42.1)	20 (80.0)	0.013 ^b^
End-of-treatment urea, mg/dL	38.5 (34.8–42.5)	71.2 (50.9–78.8)	0.003 ^c^
Urea delta, mg/dL	−5.4 (−7.9 to −2.0)	42.8 (38.0–48.6)	0.006 ^c^
End-of-treatment creatinine, mg/dL	0.93 (0.72–1.60)	2.29 (1.75–2.64)	0.003 ^c^
Creatinine delta, mg/dL	0.16 (0.10–0.57)	0.78 (0.33–1.57)	0.008 ^c^
ICU admission, *n* (%)	10 (52.6)	16 (64.0)	0.54 ^b^
Sepsis, *n* (%)	8 (42.1)	12 (48.0)	0.77 ^b^
Colistin treatment duration, days	18 (8–20)	16 (8–25)	0.79 ^c^

^a^ Independent-samples *t*-test or Mann–Whitney U test (depending on distribution). ^b^ Chi-square test or Fisher’s exact test. ^c^ Mann–Whitney U test (for median [IQR] variables).

## Data Availability

Data are available upon reasonable request.
